# Supplementary material for: A stress-induced paralog of Lhcb4 controls the photosystem II functional architecture in Arabidopsis thaliana
Source: Nat Commun. 2025 Jul 26;16:6910. doi: 10.1038/s41467-025-62085-2 (PMC12297487; doi:10.1038/s41467-025-62085-2)
Supplement: Supplementary file 1 — Supplementary Information [file 41467_2025_62085_MOESM1_ESM.pdf]

Supplementary Information

Supplementary Materials and Methods

Protein isolation and purification - The target proteins were obtained through affinity chromatography-based purification exploiting a 6X-HisTAG located (i) at the C-terminus of Lhcb4.1_TAG from the *koLhcb4 +Lhcb4.1_TAG* mutant previously described[1]; and (ii) at the Lhcb8 protein from the *koLhcb4+Lhcb8_TAG* line produced in this study. Thylakoid membranes were diluted in a 10 mM HEPES buffer at pH 7.5 to achieve a final Chl concentration of 1 mg/mL. An equal volume of 10 mM HEPES (pH 7.5) + 1.6% α-DDM was added. Solubilization was performed by incubating the solution in ice for 10 minutes. Insolubilized material was removed by centrifugation at 18,000 x g for 15 minutes at 4 °C. The purification process included IMAC using Ni-derivatized Sepharose resin, which enabled the isolation of a PSII-enriched fraction. The supernatant was incubated with Ni-derivatized resin, and purification was carried out as previously described[1]. The eluted fraction containing imidazole was subsequently loaded into sucrose density gradients containing 10 mM Hepes (pH 7.5), 0.65 M sucrose and 0.03% α-DDM. His-tagged components were fractioned by ultracentrifugation at 180,000 x g for 19 hours. Monomeric LHC proteins were isolated as previously outlined[1].

Sample preparation, data collection and processing for cryo-electron microscopy - For purification of the Lhcb8/4.1-containing PSII-SCs, the thylakoid membranes prepared from the *koLhcb4+Lhcb8_TAG* or *koLhcb4+Lhcb4.1_TAG* strains were diluted to a chlorophyll concentration of 0.5 mg/mL with 10 mM HEPES-KOH pH 7.5, and then solubilized with 0.8% α-DDM for 20 min on ice. The insoluble fraction was removed by centrifugation at 20,000 × g for 15 min. The supernatant was collected and used for SDG ultracentrifugation at 256,000 × g for 16 h at 4 °C, in a matrix containing 5–35% sucrose, 10 mM HEPES-KOH pH 7.5, 0.5 M Betaine and 0.02% α-DDM. The bands containing PSII-SCs were collected, concentrated in an Amicon Ultra-15 centrifugal filter unit of 100-kDa molecular weight cutoff (Merck Millipore), diluted tenfold with a sucrose-free buffer (10 mM HEPES-KOH pH 7.5, 0.5 M Betaine and 0.02% α-DDM) and concentrated back to the volume before dilution. The wash procedure was repeated thrice, and the samples were concentrated to a final chlorophyll concentration at 8 mg/mL.
For cryo-EM grid preparation, 3 μL of the concentrated sample was loaded onto the H_2/O_2 glow-discharged holey carbon grid (Quantifoil 300-mesh, R1.2/1.3). The grid was blotted for 7 s with a force level of 0 at 4 °C and humidity of 100%, flash-frozen into liquid ethane using Vitrobot Mark IV (Thermo Fisher Scientific) and then transferred to liquid nitrogen for storage. Micrographs were collected by using the SerialEM software suite[2] on a 300-kV Titan Krios electron microscope (Thermo Fisher Scientific) equipped with a K3 Summit direct electron detector (Gatan). For the Lhcb8-containing PSII-SC samples, two sets of data (2,708 and 2,556 micrographs, respectively) were collected at a defocus range of -1 to -2 μm, with a pixel size of 0.53 Å in the super resolution mode. The images were recorded by using the beam-image shift data collection method[3]. The total dose of each micrograph was 60 $e^-/Å^2$ and was fractionated into 32 frames. For the Lhcb4.1-containing PSII-SC sample, a total of 3,004 micrographs were collected using the same parameters as those for collecting the Lhcb8-containing PSII-SC data. A summary of the statistics for data collection is provided in Supplementary Table S3.
The cryo-EM data were processed by using CryoSPARC v.4.3.1[4]. The patch-based motion correction corrected the imported movie stack and dose-weighted with 2-fold binning. The motion-corrected micrograph's contrast transfer function (CTF) parameters were estimated using CTFFIND4.1[5]. For better particle-picking performance, the template-based particle-picking procedure was used, and the templates came from the well-defined class images of 2D classification with the initial particle images obtained through the manual picking process. For the second dataset of the Lhcb8-C_2S_2 PSII SC, the cryo-EM map obtained after the non-uniform refinement from the first dataset was used to create more accurate and diverse templates. Subsequently, the particles were extracted for further 2D classification, ab-initio reconstruction, heterogeneous refinement, global/local CTF refinement, and non-uniform refinement. The detailed data-processing workflow is shown in Supplementary Fig. S6 and 7a. To improve the densities of

Lhcb8/4.1 and other peripheral antenna complexes, 3D classification and local refinement using the local masks of Lhcb8/4.1-CP47 and Lhcb8/4.1-LHCII-Lhcb5 were performed after C2 symmetry expansion. Masks for 3D classifications and local refinements were generated with UCSF Chimera v.1.16[6] and modified by using the volume tool in CryoSPARC. The whole map and two local maps were sharpened and provided for generating the composite map in Phenix v.1.20[7] using the Combine Focused Maps program. The data for generating the gold standard Fourier shell correlation (GSFSC) curves and three-dimensional orientation distribution plots were exported from CryoSPARC (Supplementary Fig. S7b, d and e). The local resolution of the maps was estimated by using CryoSPARC and Phenix (Supplementary Fig. S7f).
For model building, the previously-reported *A. thaliana* $C_2S_2M_2$ PSII-SC model (PDB ID: 7OUI; https://doi.org/10.2210/pdb7OUI/pdb) with M-LHCII and CP24 removed was used as the initial C_2S_2 model. The initial model was docked into the final composite map with UCSF Chimera and manually adjusted in Coot v.0.9.8[8]. In the cryo-EM map of Lhcb8-C_2S_2, the model of Lhcb8 was rebuilt from the previous model of Lhcb4.1 by referring to the amino acid sequence of Lhcb8. The monomer A of S-LHCII trimer facing CP43 was rebuilt as Lhcb2.2 in both Lhcb8-C_2S_2 and Lhcb4.1-C_2S_2 maps according to the characteristic local density features. Several other errors in the previous model, such as chlorophyll-ligand coordination bonds, loop registration and carotenoid configuration, have been identified and fixed in the structure models of Lhcb8-C_2S_2 and Lhcb4.1-C_2S_2. The manually adjusted models were further subjected to the real-space refinement in Phenix v.1.20, and the geometric restraints for the co-factors and chlorophyll-ligand coordination bond parameters were supplied during the refinement process. The automatic real-space refinement and manual inspection processes were carried out iteratively until the geometries of the final structures converged to a reasonable range, as assessed by MolProbity[9]. The statistics for data collection and structure refinement are summarized in Supplementary Table S3. The high-resolution images illustrating the structures were prepared using ChimeraX v.1.8 and Pymol v.2.5.4 (Schrödinger, LLC).

Phylogenetic reconstruction and multiple sequence alignment - The following gene identifier sequences were included in the phylogenetic reconstruction analysis: Ostreococcus lucimarinus: eugene.1500010213; Micromonas sp RCC299: estExt_Genewise2.C_Chr_150046; *Volvox carteri*: Vocar.0021s0124; *Chlamydomonas reinhardtii*: Cre17.g720250; *Ginkgo biloba*: GBI00028088; *Pinus sylvestris*: PSY00025573; *Picea glauca*: PGL00023147; *Prunus persica*: Prupe.2G248900; *Glycine max Wm82.a2*: Glyma.18G028400; *Eucalyptus grandis*: Eucgr.G03060; *Manihot esculenta*: Manes.07G128500; *Gossypium raimondii*: Gorai.011G285900; *Arabidopsis thaliana*: AT2G40100; *Azolla filiculoides*: s0173.g055756; *Chara braunii*: CBR_g9133; *Physcomitrium patens*: Pp3c12_25460; *Marchantia polymorpha*: Mapoly0001s0025; *Selaginella moellendorffii*: 149722; *Anthoceros punctatus*: utg000103l.83.1; *Hordeum vulgare*: HORVU5Hr1G013200; *Triticum aestivum*: Traes_5BS_B3AB099FF; *Prunus persica*: Prupe.6G276200; *Solanum lycopersicum*: Solyc09g014520.3; *Glycine max Wm82.a2*: Glyma.01G115900; *Gossypium raimondii*: Gorai.002G263900; *Manihot esculenta*: Manes.08G091700; Eucalyptus grandis: Eucgr.K02983; Musa acuminata: GSMUA_AchrUn_randomG28100_001; *Arabidopsis thaliana*: AT3G08940; Brassica oleracea capitata: Bol012796; Arabidopsis thaliana: AT5G01530; *Spirodela polyrhiza*: Spipo22G0045300; *Aquilegia coerulea*: Aqcoe2G097400; *Amborella trichopoda*: evm_27.TU.AmTr_v1.0_scaffold00029.405; *Triticum aestivum*: Traes_2AS_8169B126A; *Oryza sativa*: LOC_Os07g37240; *Zea Mays*: GRMZM2G033885_T01; *Setaria italica*: Seita.2G351600. Species and accession code used for the C-terminus alignment are listed the following: *Tetraselmis sp. RG-15*: AAB70556.1; *Ostreococcus tauri*: AAY27543.1; *Micromonas pusilla*: XP_003063531.1; *Monoraphidium neglectum*: XP_013903947.1; *Bryopsis sp. KO-2023*: GMH44747.1; *Auxenochlorella protothecoides*: XP_011400395.1; *Acetabularia acetabulum*: DAA05897.1; *Ulva prolifera*: QOJ43403.1; *Tetradesmus obliquus*: WIA19698.1; *Haematococcus lacustris*: KAJ9515268.1; *Chlamydomonas incerta*: KAG2437880.1; *Pleodorina starrii*: GLC43598.1.

Supplementary Figures

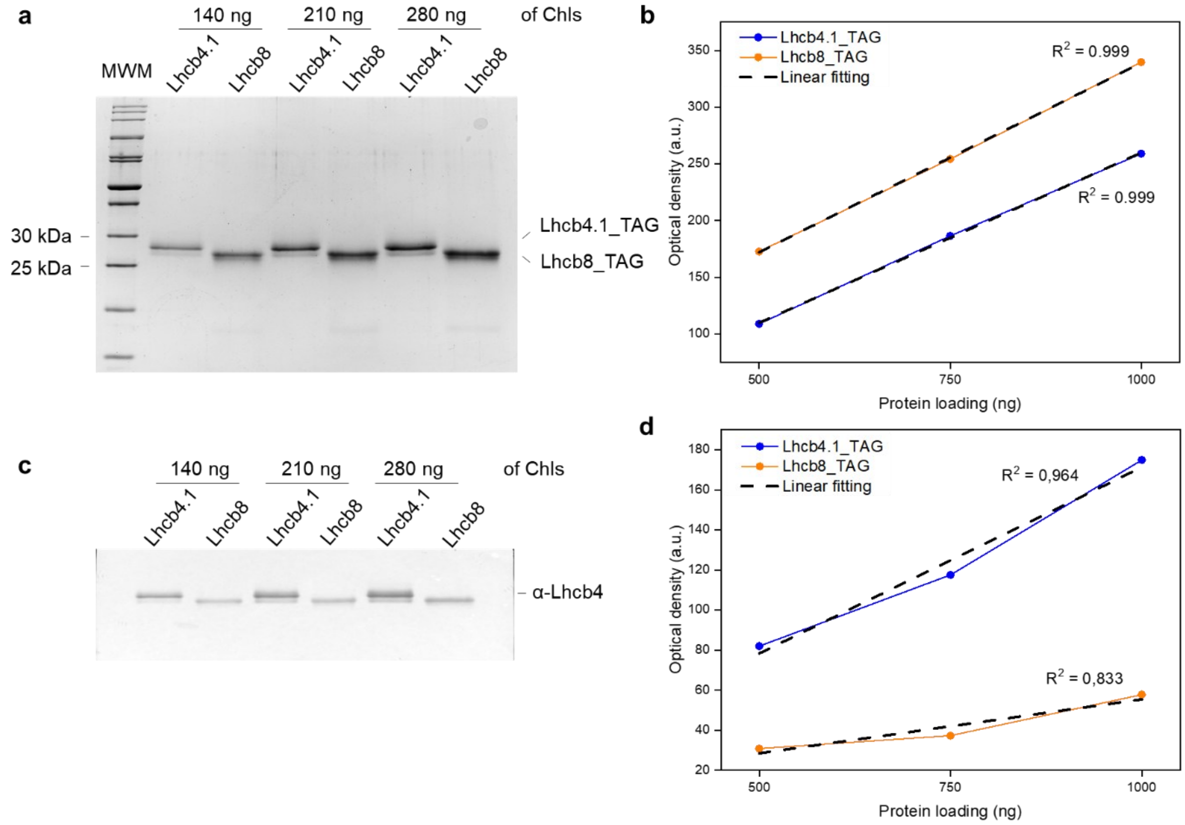

Supplementary Fig. S1 α-Lhcb4 antibody reactivity test. **a** Coomassie-stained SDS-PAGE loaded with three different amounts of purified Lhcb4.1_TAG and Lhcb8_TAG proteins. **b** Linear correlations assessed between the protein amounts and the densitometric estimation of purified Lhcb4.1_TAG and Lhcb8_TAG fractionated by SDS-PAGE. **c** Immunodecoration of nitrocellulose membrane developed with the α-Lhcb4 primary antibody. Samples included three different amounts of each purified protein. **d** Linear correlations assessed between the densitometric values from panel c and the amounts of purified proteins loaded. The reactivity of the α-Lhcb4 against Lhcb8_TAG was approximately 4-fold lower with respect to that against Lhcb4.1_TAG. Proteins were purified from plants generated in this work (see Methods).

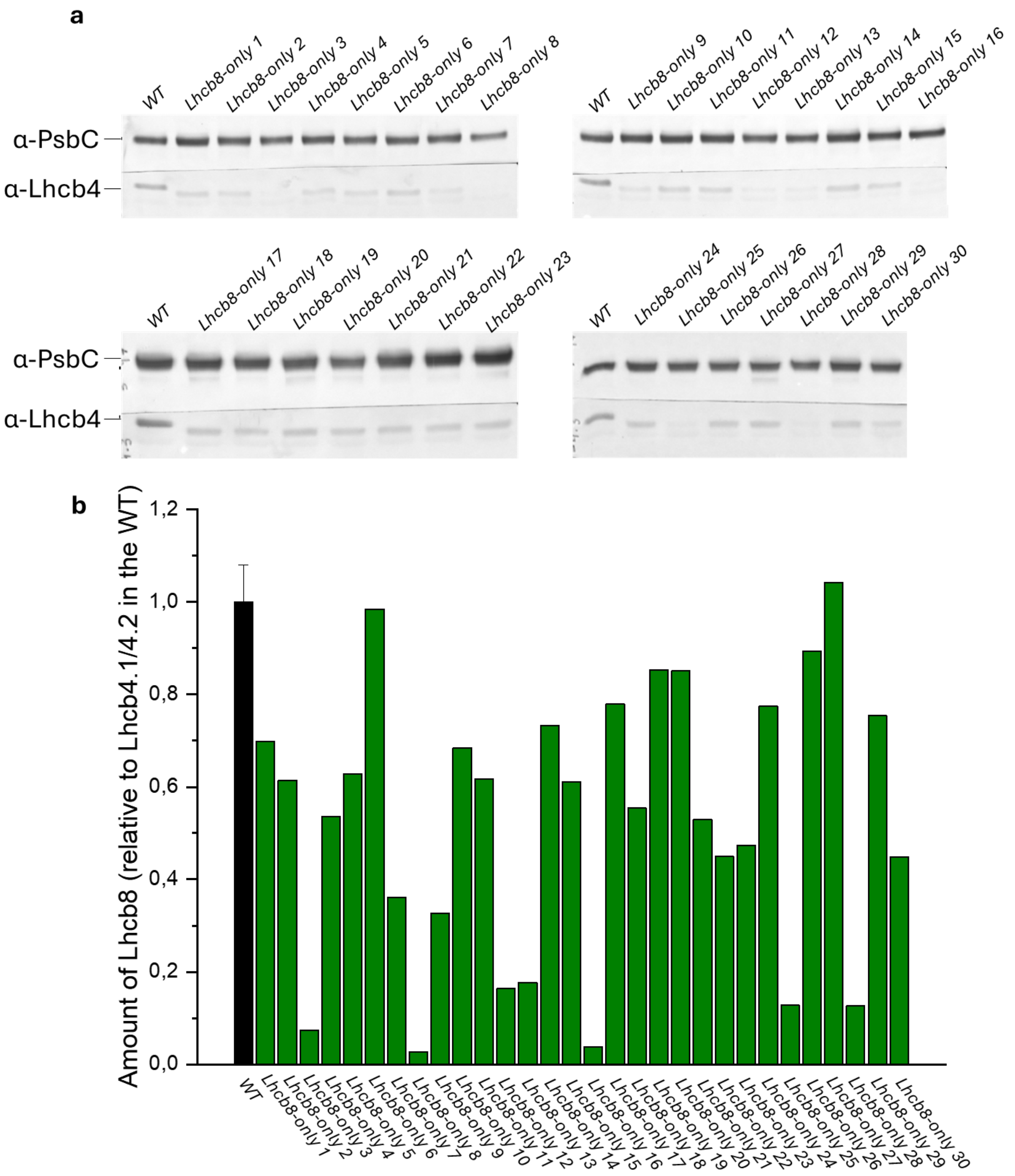

Supplementary Fig. S2 Estimation of Lhcb8 protein abundance in Lhcb8-expressing T1 lines. **a** Western blot analysis carried out on leaf protein extracts from 30 independent T1 lines using α-Lhcb4 and α-PsbC antibodies. **b** Densitometric analysis of Lhcb8 content in T1 lines screened by western blot. The optical densities of Lhcb8 bands were normalized to the core antenna PsbC content and adjusted for the 4-fold lower affinity of α-Lhcb4 against Lhcb8 (see Supplementary Fig. S1).

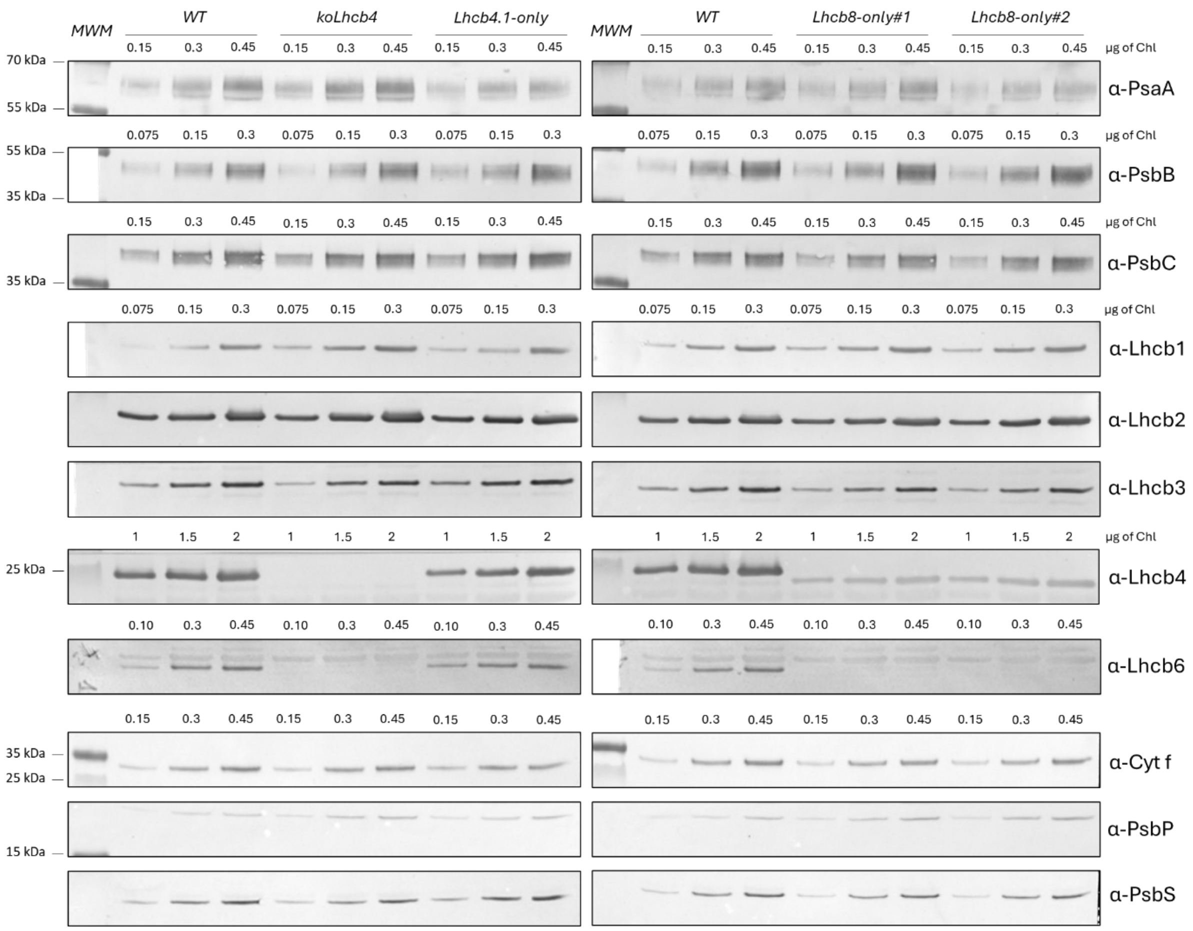

Supplementary Fig. S3 Western blot analysis on thylakoid membranes from *Lhcb8-only* lines and control genotypes. Gels were loaded with three different amounts of thylakoid extracts and immunotitrated against some major subunits of the photosynthetic apparatus. (*MWM*: molecular weight marker).

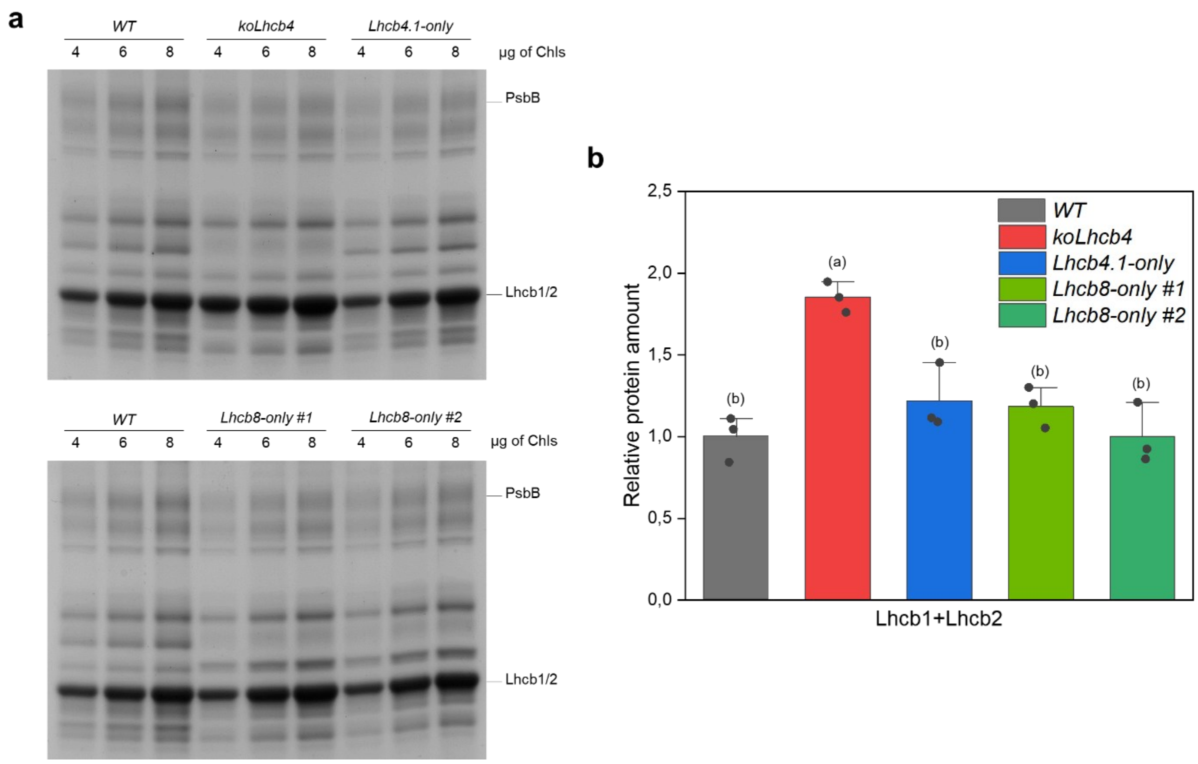

Supplementary Fig. S4 Biochemical characterization of selected genotypes. **a** Coomassie-stained SDS-PAGE loaded with three different amounts of thylakoid extracts (corresponding to 4, 6 and 8 µg of Chl). **b** Densitometric analysis of Lhcb1/Lhcb2 content. Values were normalized to the PSII core (PsbB content) and expressed relative to the corresponding WT value. Data are reported as mean ± standard deviation of n = 3 technical replicates. Letters in brackets represent the result of Tukey's test (P-value ≤ 0.05).

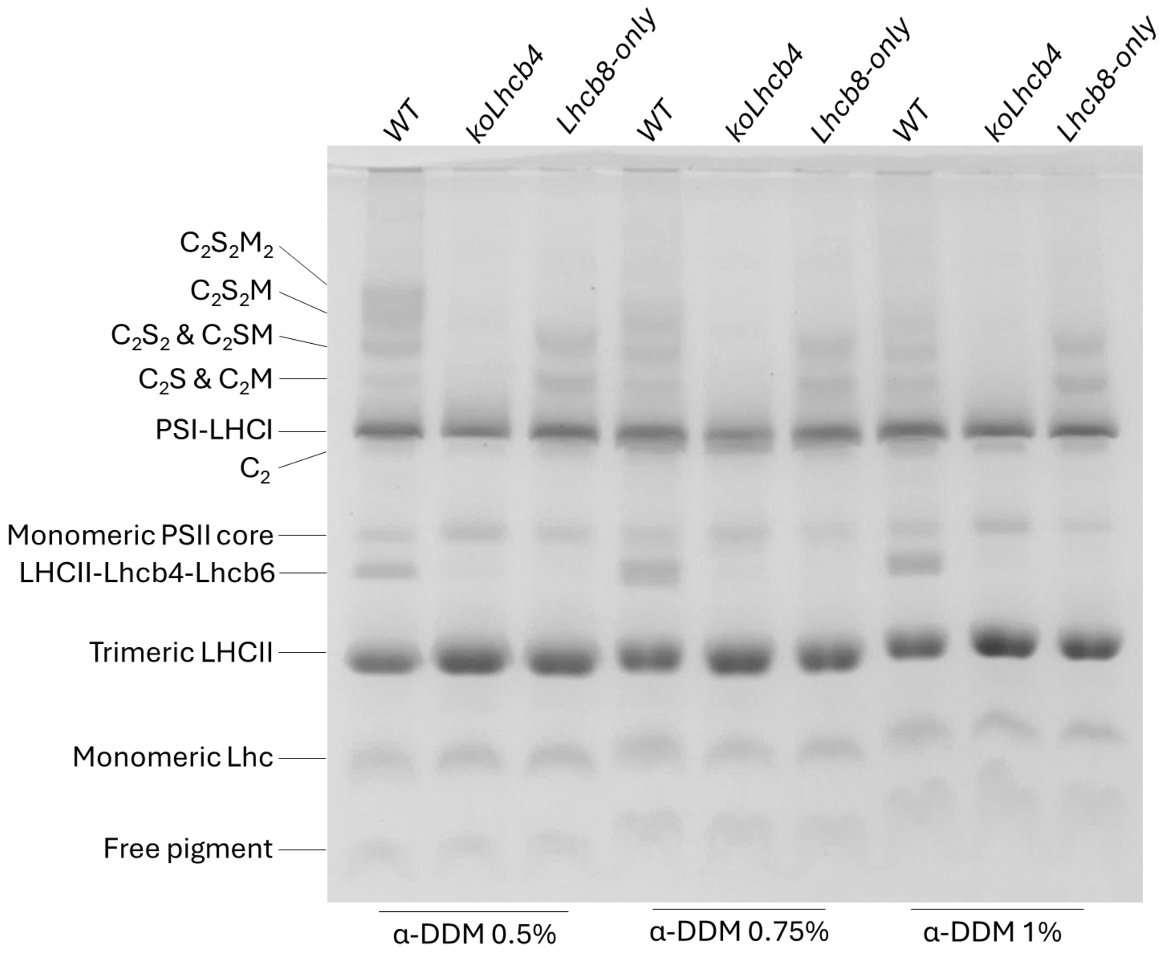

Supplementary Fig. S5 Organization of thylakoid pigment-protein complexes. Photosynthetic membranes from *wild type*, *koLhcb4* and *Lhcb8-only* plants were separated by non-denaturing Deriphat-PAGE (4%-12% acrylamide), to examine the overall Chl-binding protein organisation and the PSII supercomplex stability at increasing detergent concentrations. Solubilisation of thylakoid membranes was performed with 0.5%, 0.75% and 1% α-DDM. The composition of each band is indicated based on earlier work[10].

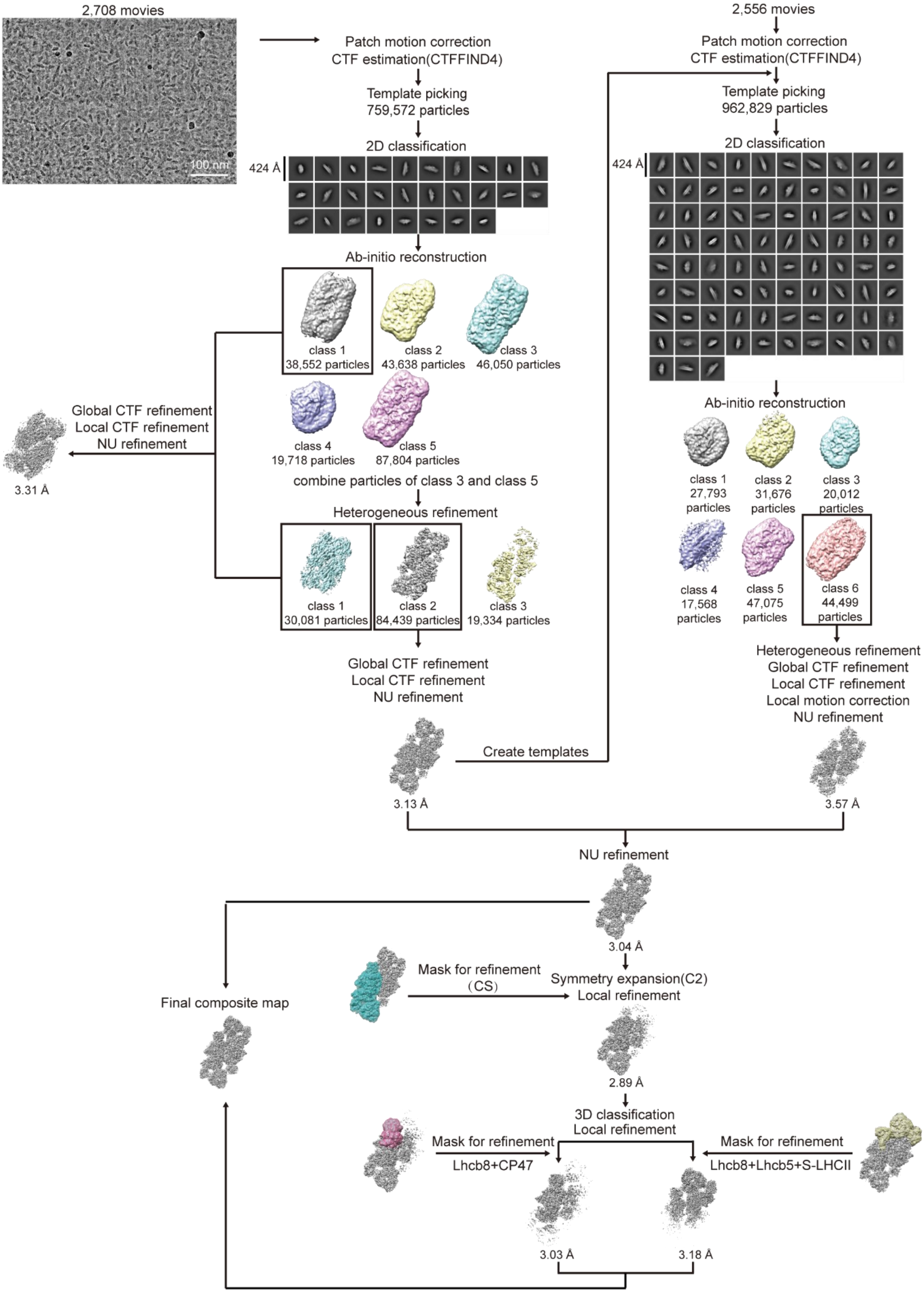

Supplementary Fig. S6 Overall scheme of cryo-EM data processing for the Lhcb8-C_2S_2 supercomplex.

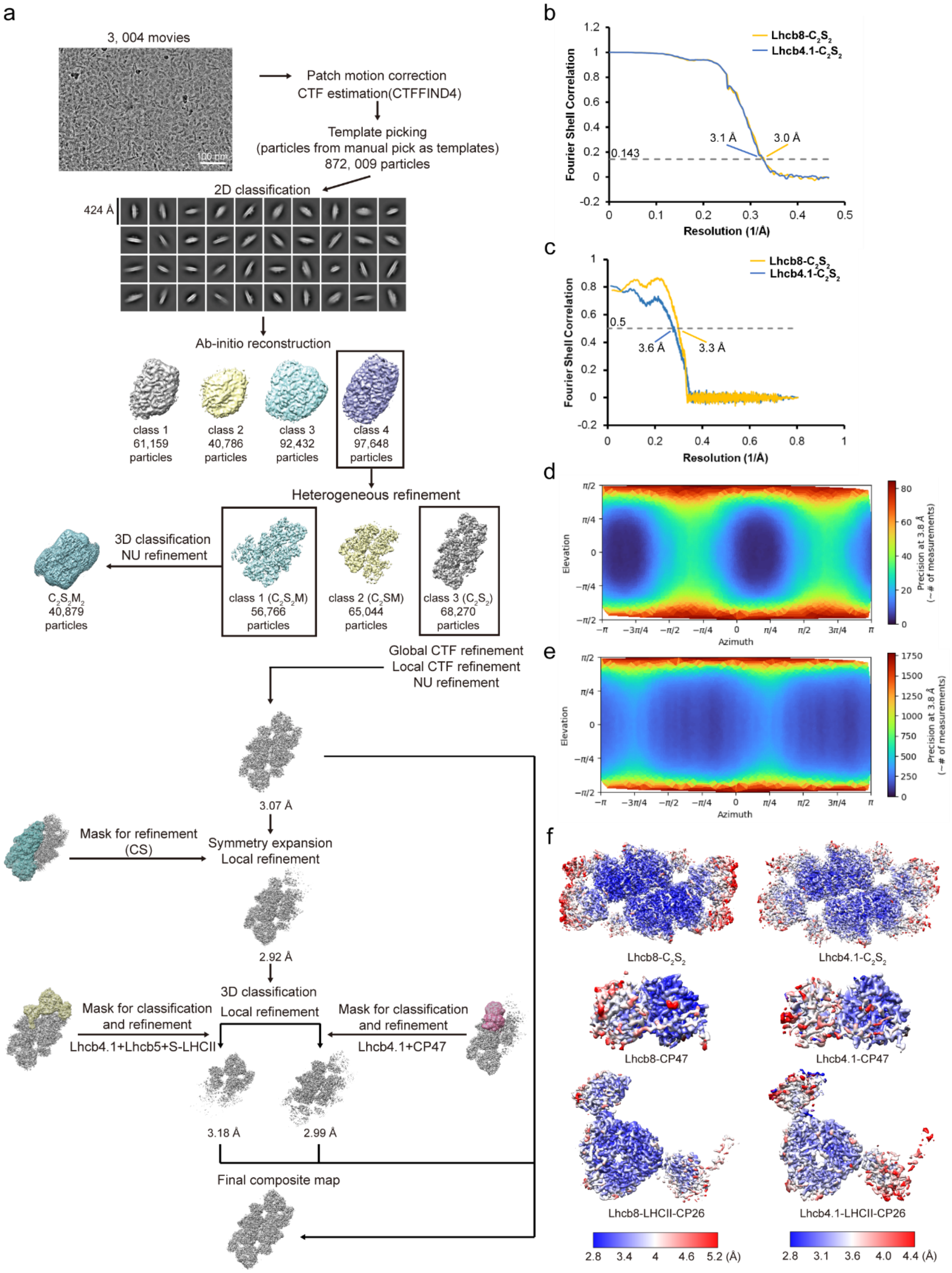

Supplementary Fig. S7 Overall scheme of cryo-EM data processing of the Lhcb4.1-C_2S_2 supercomplex and evaluation of the data, maps and models of Lhcb4.1-C_2S_2 and Lhcb8-C_2S_2. **a** The overall scheme for the single particle cryo-EM data processing procedure of the Lhcb4.1-C_2S_2 supercomplex. The circled 3D classes are chosen for further refinements. **b** The gold standard Fourier shell correlation (GSFSC) curve of the Lhcb8-C_2S_2 and Lhcb4.1-C_2S_2 maps with a resolution-cutoff threshold at 0.143. **c** The Fourier shell

correlation (FSC) curves between the structural model and the composite cryo-EM density map of the Lhcb8-C_2S_2 and Lhcb4.1-C_2S_2 supercomplexes. **d** and **e** Representative orientational distribution plots for the particle sets of the Lhcb8-C_2S_2 **d** and Lhcb4.1-C_2S_2 **e** supercomplexes respectively. **f** Estimation of the local resolution in the cryo-EM maps of the Lhcb8-C_2S_2 and Lhcb4.1-C_2S_2 supercomplexes.

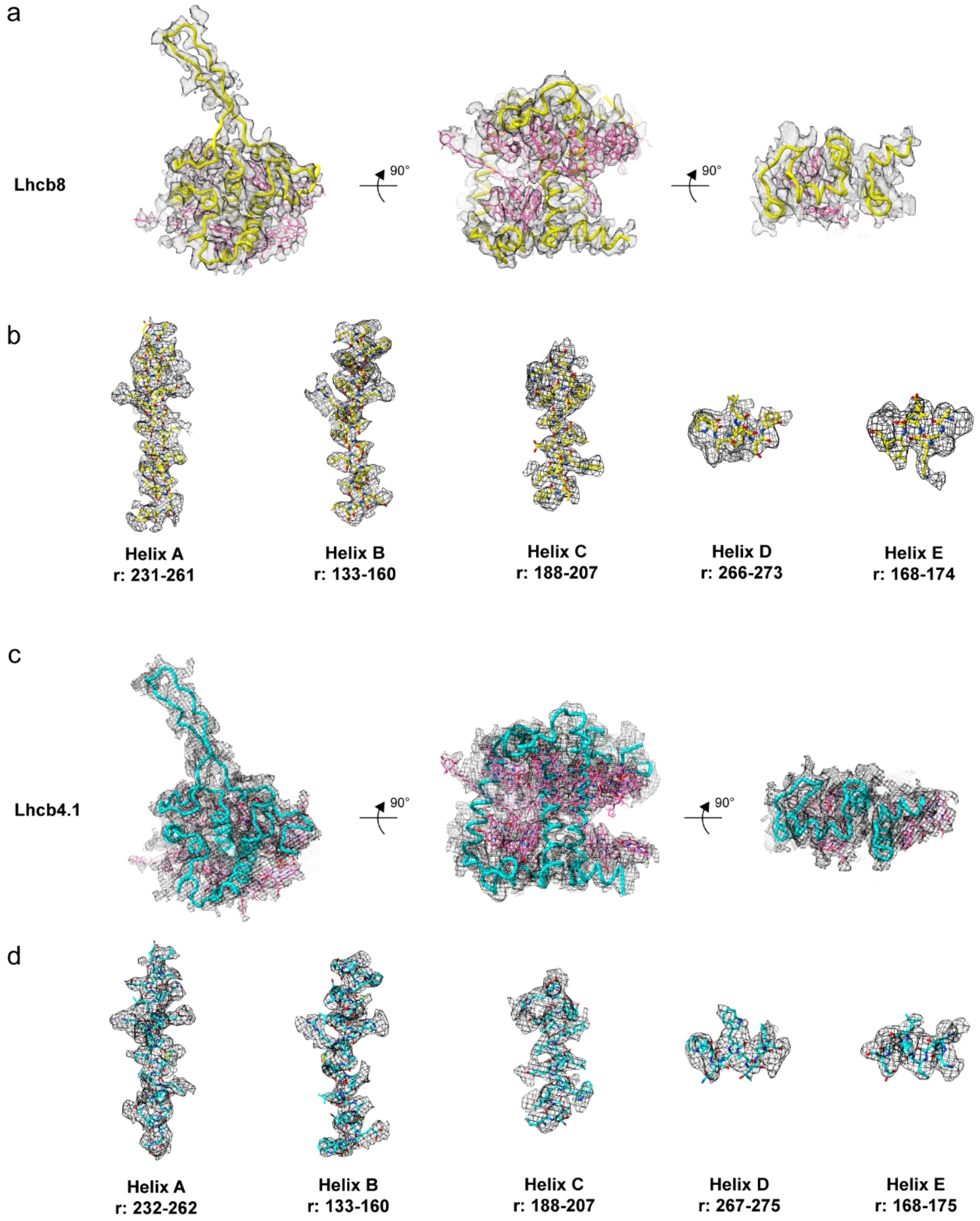

Supplementary Fig. S8 Cryo-EM densities of Lhcb8 and Lhcb4.1. **a** and **b** The overall cryo-EM density of Lhcb8 **a** and the five helices in its structure **b**. **c** and **d** The overall cryo-EM density of Lhcb4.1 **c** and the five helices in its structure **d**. The cartoon models of Lhcb8 and Lhcb4.1 apoproteins are colored yellow and cyan, respectively. The ligands are presented as stick models in pink.

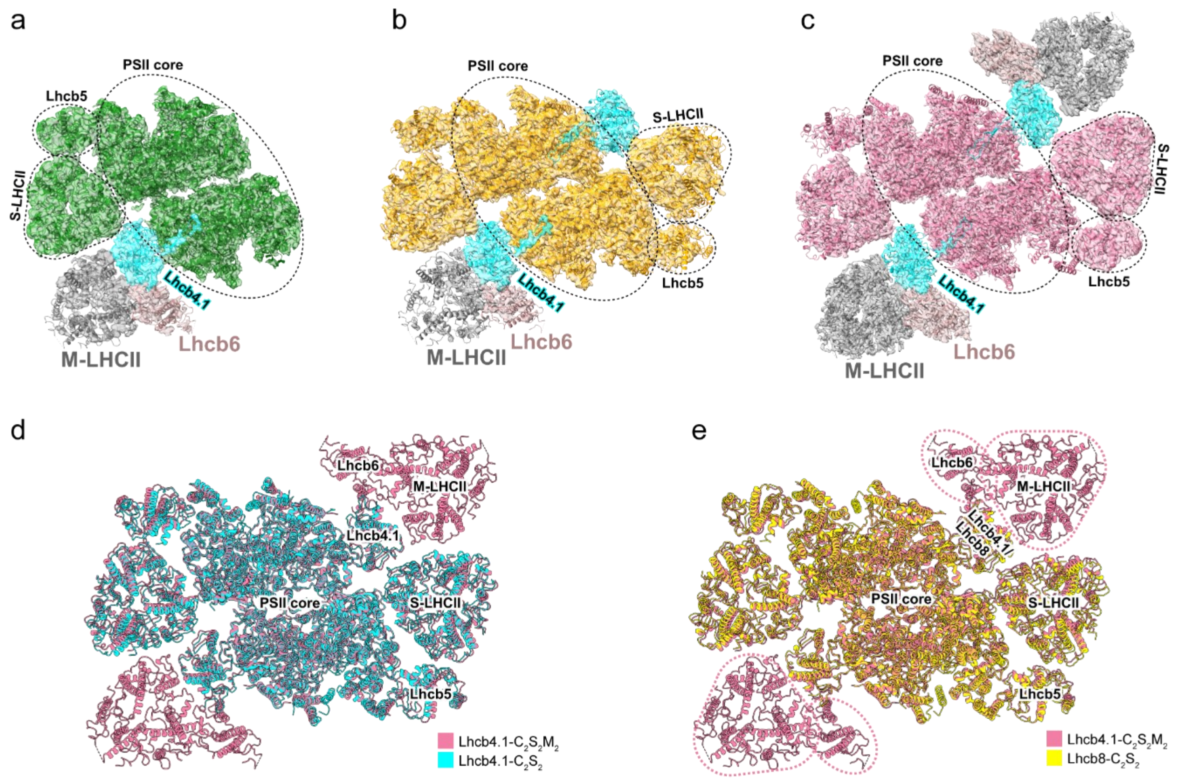

Supplementary Fig. S9 The C_2SM, C_2S_2M and $C_2S_2M_2$ supercomplexes from the *Lhcb4.1-only* plant. **a**-**c** The overall architectures of C_2SM, C_2S_2M and $C_2S_2M_2$-type PSII-SCs from the *Lhcb4.1-only* plants. The cryo-EM maps are superposed with the corresponding cartoon models. Lhcb6 and M-LHCII are colored in brown and grey, respectively. Note that the densities of M-LHCII and Lhcb6 are relatively weaker than those of S-LHCII and C_2 regions presumably due to high mobility or low occupancy. **d** Superposition of Lhcb4.1-C_2S_2 with Lhcb4.1-$C_2S_2M_2$. **e** Superposition of Lhcb8-C_2S_2 with Lhcb4.1-$C_2S_2M_2$. The dashed elliptical rings in e indicate the vacant Lhcb6 and M-LHCII-binding sites in Lhcb8-C_2S_2.

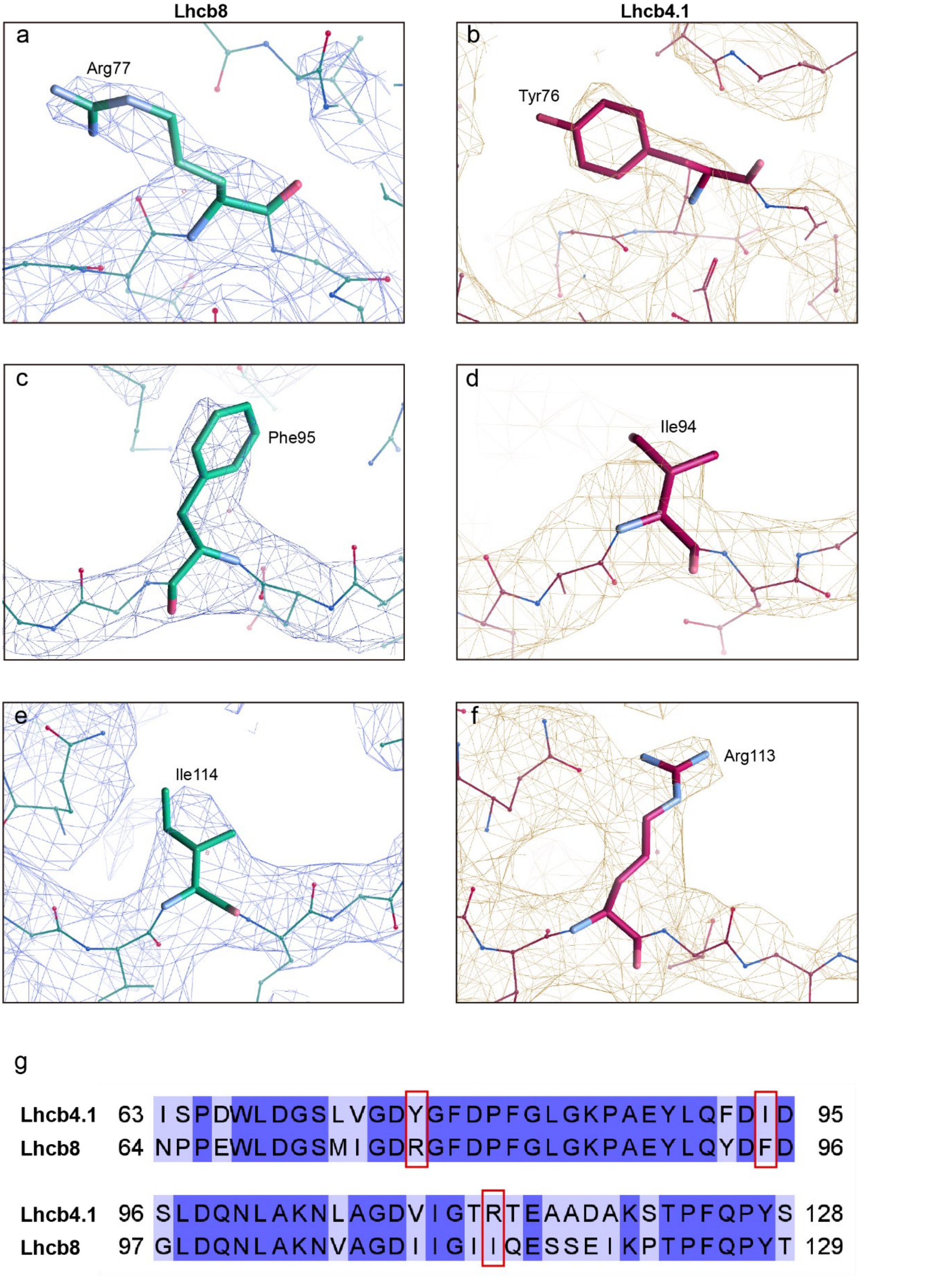

Supplementary Fig. S10 The characteristic cryo-EM density features of Lhcb8 and Lhcb4.1. **a**-**f** The local cryo-EM density features distinguishing Lhcb8 from Lhcb4.1. **g** The amino acid sequence alignment of Lhcb8 and Lhcb4.1 in a local region bearing distinct features corresponding to those shown in a-f.

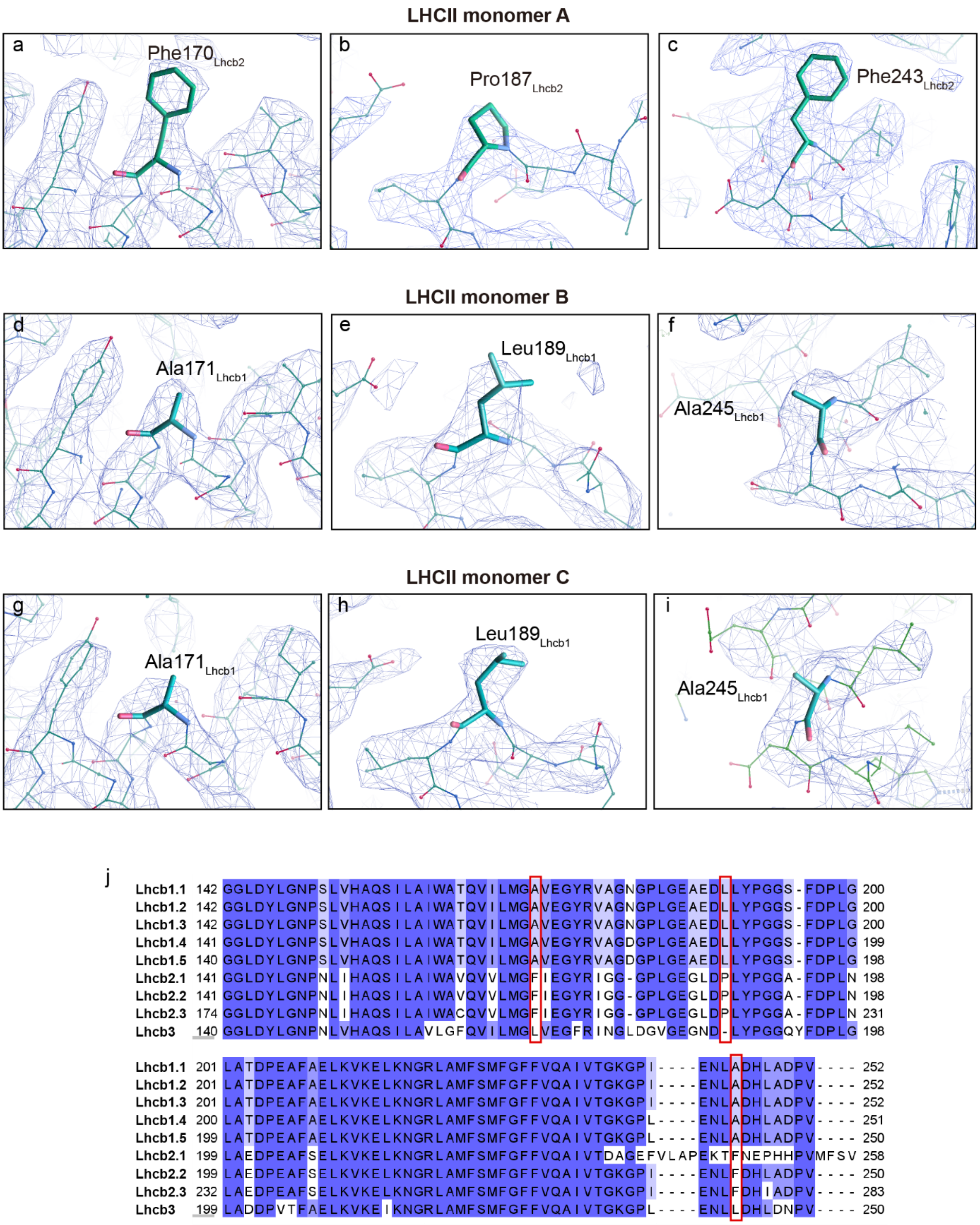

Supplementary Fig. S11 The local cryo-EM densities characteristic of Lhcb1 and Lhcb2 in the S-LHCII trimer. **a**-**c** The local cryo-EM densities of three characteristic amino acid residues for the LHCII monomer A assigned as Lhcb2. The structural model is constructed with the amino acid sequence of Lhcb2.2. **d**-**i** The cryo-EM densities of the corresponding three amino acid residues for the other two LHCII monomers (B and C) assigned as Lhcb1. The structural models of both monomers B and C were constructed with the amino acid sequence of Lhcb1.1. **j** The amino acid sequence alignment of Lhcb1-3 in a local region bearing three distinct features corresponding to those shown in **a**-**i**.

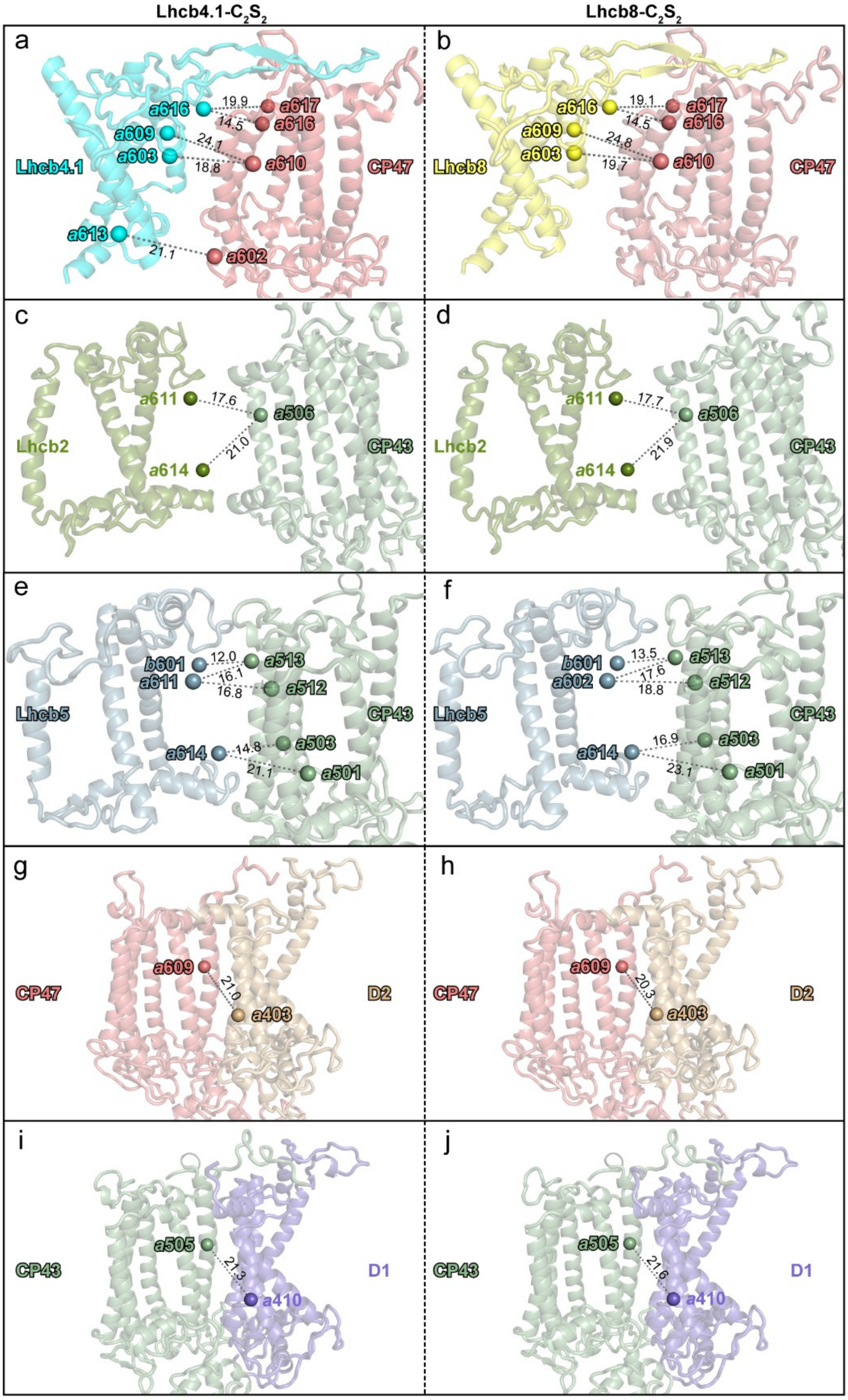

Supplementary Fig. S12 The interfacial Chl molecules between adjacent antenna complexes or between the core antenna and D1/D2 in Lhcb4.1-C_2S_2 and Lhcb8-C_2S_2. **a**-**f** The interfacial Chl pairs between Lhcb4.1/Lhcb8 and CP47, between Lhcb2 and CP43 and between Lhcb5 and CP43 in Lhcb4.1-C_2S_2 **a**, **c** and **e** and Lhcb8-C_2S_2 **b**, **d** and **f**. **g-j** The Chl pairs connecting CP47 with D2 and CP43 with D1 in Lhcb4.1-C_2S_2 **g** and **i** and Lhcb8-C_2S_2 **h** and **j**.

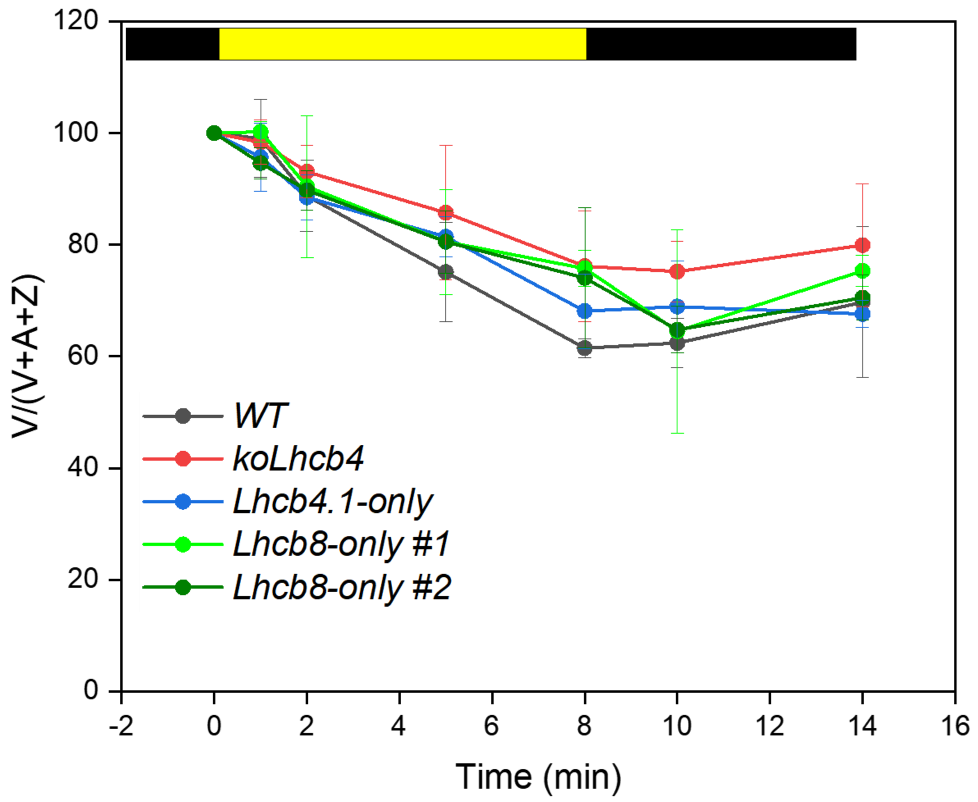

Supplementary Fig. S13 Kinetics of violaxanthin de-epoxidation in *Lhcb8-only* and control genotypes. Leaf discs (dark-adapted for 4 hours) were exposed to high light (1,280 µmol photons m^{-2} s^{-1}) for 8 min, followed by 6 min of dark recovery. At different time points, leaf discs were frozen in liquid nitrogen, and pigments were extracted for HPLC analysis. Yellow and black bars represent light and dark periods, respectively. The de-epoxidation index was calculated by dividing Violaxanthin's picomoles by the total amount of xanthophylls' picomoles (Violaxanthin+Antheraxanthin+Zeaxanthin) and normalized to 100 at time zero. Data are expressed as mean ± standard deviation of n = 3 biological replicates.

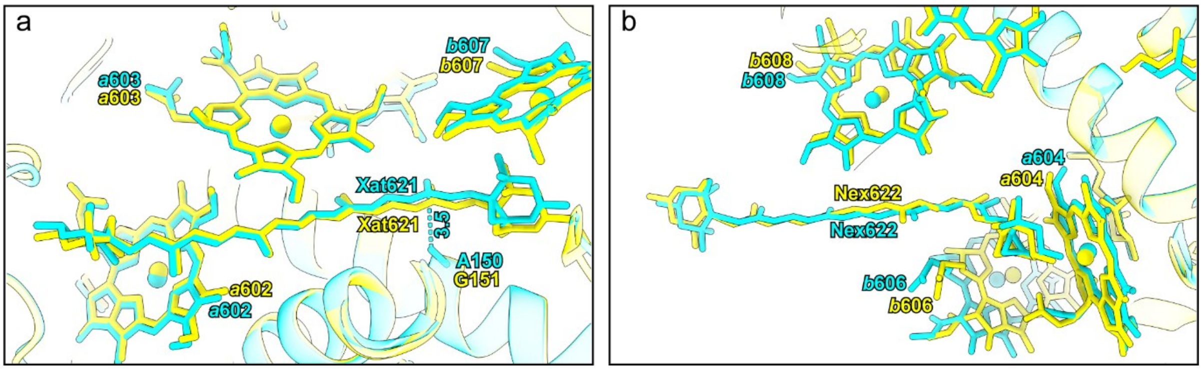

Supplementary Fig. S14 Superposition of violaxanthin and neoxanthin binding sites from Lhcb8 and Lhcb4.1. **a** Xat621 (violaxanthin at the 621 site) and its surrounding groups. **b** Nex622 (neoxanthin at the 622 site) and its nearby pigments. Color codes: yellow, Lhcb8; cyan, Lhcb4.1.

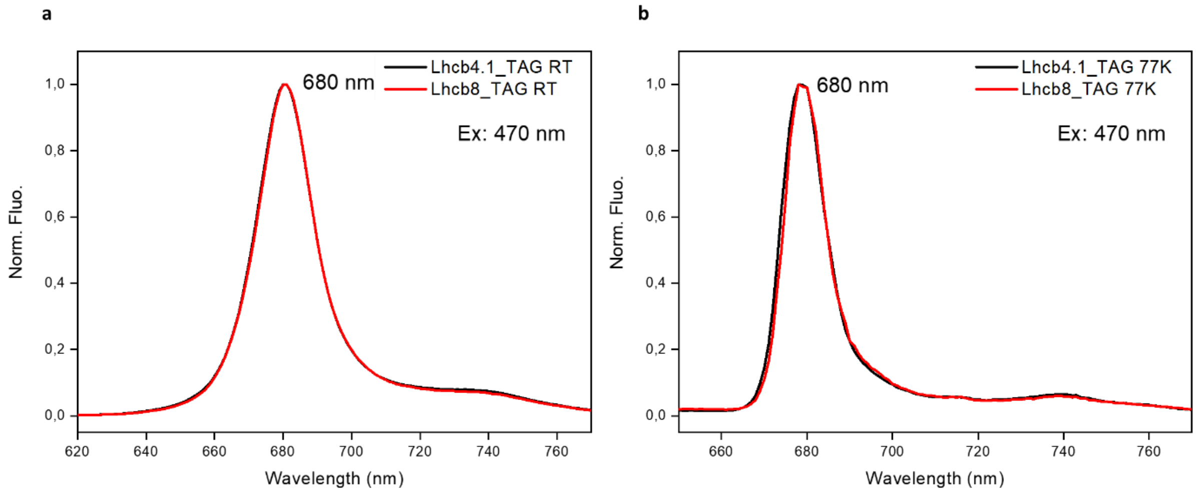

Supplementary Fig. S15 Steady-state fluorescence emission on isolated proteins. **a** Steady-state fluorescence emission of isolated Lhcb4.1_TAG and Lhcb8_TAG holoproteins at room temperature upon excitation at 470 nm. **b** Steady-state fluorescence emission of isolated Lhcb4.1_TAG and Lhcb8_TAG holoproteins at 77 K upon excitation at 470 nm.

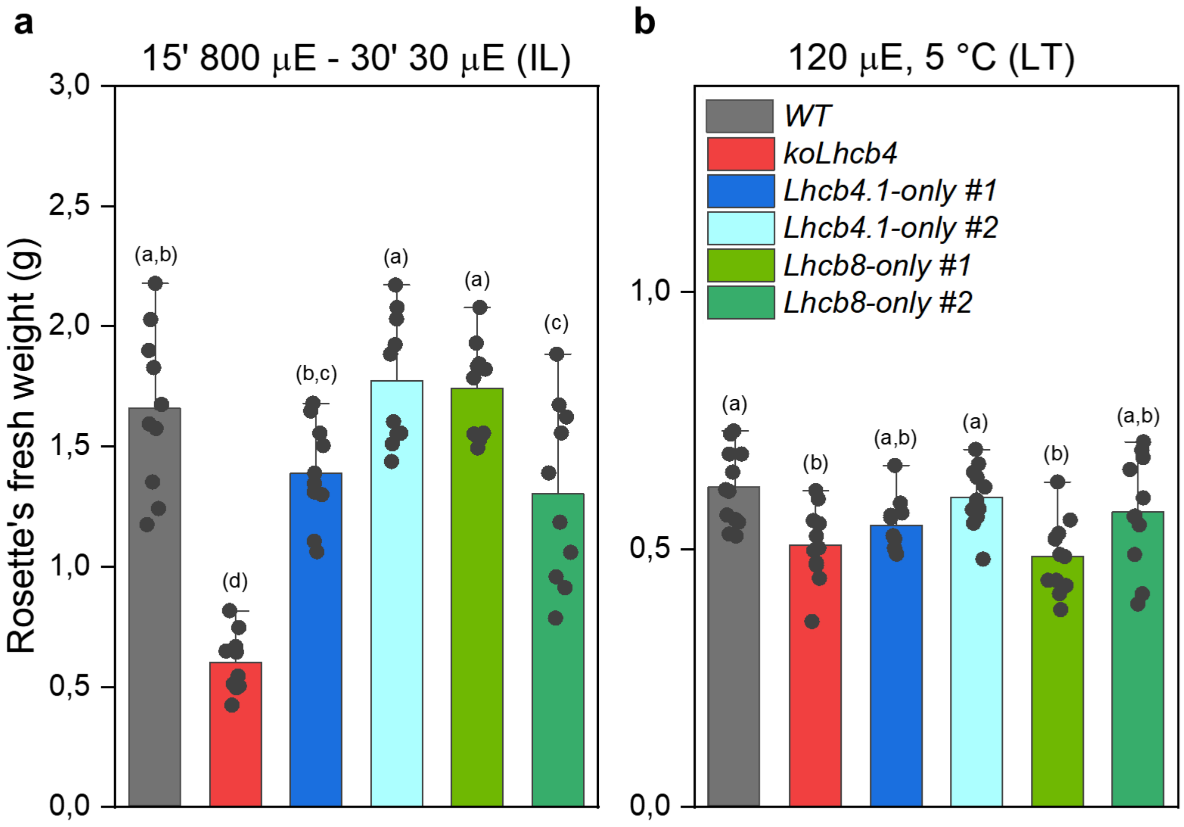

Supplementary Fig. S16 Growth trial of selected genotypes in abiotic stress conditions. **a** Fresh weight of plant rosettes harvested after 2 weeks of growth under control light conditions and 4 weeks under intermittent light conditions (15 minutes at 800 µmol photons m^{-2} s^{-1} and 30 minutes at 30 µmol photons m^{-2} s^{-1}, 23 °C, 70% relative humidity, 8/16 h of day/night). **b** Fresh weight of plant rosettes harvested after 2 weeks of growth under control temperature conditions (23 °C) and then 12 weeks at 5 °C (120 µmol photons m^{-2} s^{-1}, 5 °C, 70% relative humidity, 8/16 h of day/night). Data are shown as mean ± standard deviation of n = 10 biological replicates. The statistical significance was determined by a one-way ANOVA test followed by the Tukey's test and depicted with lower-case letters (P-value ≤ 0.05). The experiment was repeated 2 time with similar results.

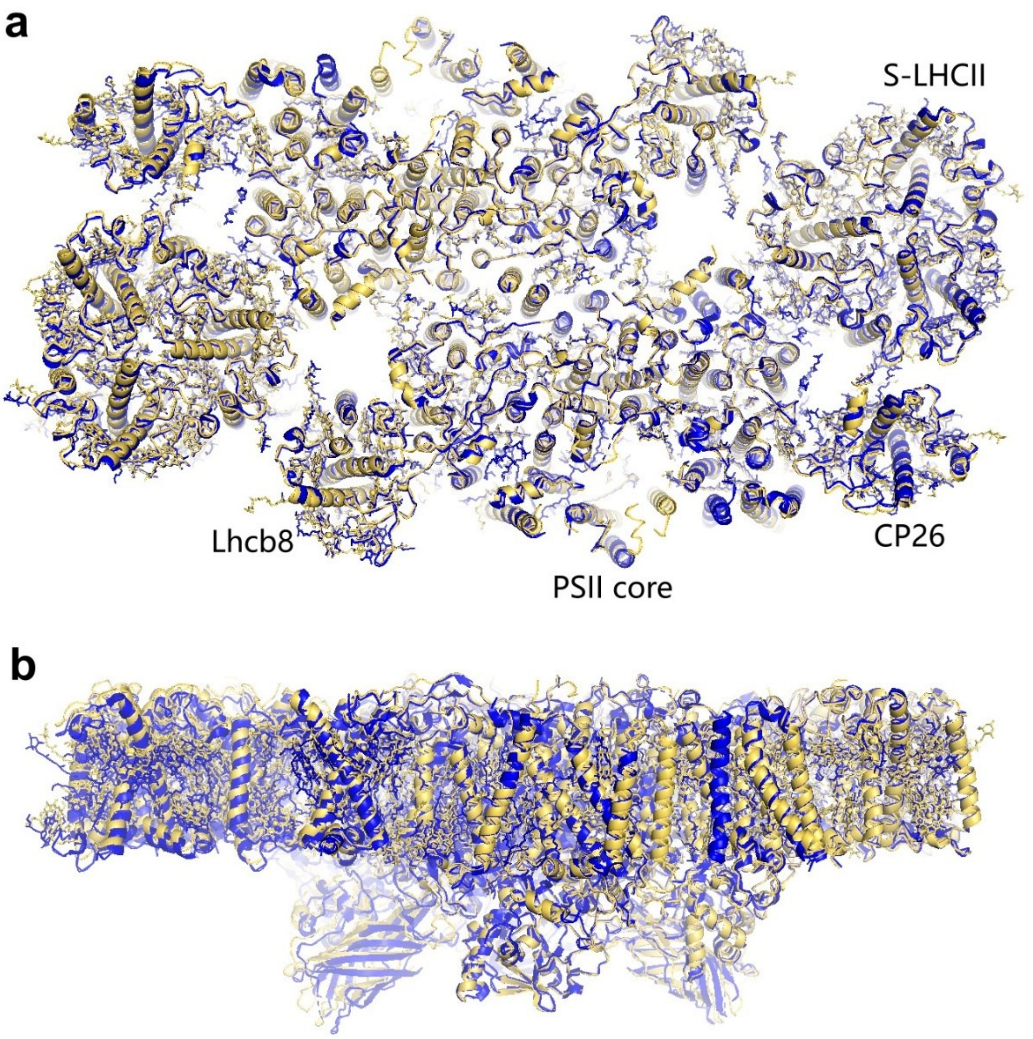

Supplementary Fig. S17 Superposition of Lhcb8-C_2S_2 from *A. thaliana* with the one from *P. abies*. **a** Top view; **b** Side view. The two supercomplexes are superposed on the D1 proteins of one PSII monomer. Color code: golden, Lhcb8-C_2S_2 from *A. thaliana*; blue, Lhcb8-C_2S_2 from *P. abies* (PDB code: 8C29). The proteins are shown as cartoon models, while the pigments are presented as stick models. Note that the S-LHCII from *P. abies* Lhcb8-C_2S_2 is a homotrimer of Lhcb1, whereas the one in *A. thaliana* Lhcb8-C_2S_2 is a $(Lhcb1)_2$Lhcb2 heterotrimer.

Supplementary Table S1

	Neoxanthin	*Violaxanthin*	*Lutein*	*Chl b*	*Chl a*
Lhcb4.1_TAG	0,91 ± 0,07	1,03 ± 0,01	1,04 ± 0,01	3,87 ± 0,04	9,72 ± 0,16
Lhcb8_TAG	0,92 ± 0,03	0,99 ± 0,03	1,07 ± 0,03	2,98 ± 0,03	8,02 ± 0,14

Pigment composition of Lhcb4.1_TAG and Lhcb8_TAG holoproteins. Pigment content was quantified by HPLC analysis, and the values were normalised to the three carotenoids. Data are expressed as mean ± standard deviation of n = 3 technical replicates.

Supplementary Table S2

Primer name	Primer sequence
SbfIProm4.1FW	5' - ttcctgcaggTCGGAGATCGATTTGGAGAG - 3'
Prom4.1RV	5' - tagccatattCTCCGGCTAATTGGGTTTTGTG - 3'
Lhcb4.3FW	5' - ttagccggagAATATGGCTACCACCACTGC - 3'
Lhcb4.3RVPacI	5' - ccttaattaaTCACGGTATATTAGTTTAGAACATAAAC - 3'
Lhcb4.3TAG**C**FW	5'- catcatcaccaccacTAGTTAATGAACCTTTCTATCTTTATTTTATC - 3'
Lhcb4.3TAG**C**RV	5'- atgaccacctcctccATTGTTAAAGGTGGCAAG - 3'

Sequences of oligonucleotides used for cloning the Lhcb8 CDS downstream of the Lhcb4.1 promoter, and for the addition of the poly-His affinity tag (6xHisTAG, see Methods for details).

Supplementary Table S3

**Data collection and processing**	**Lhcb8-C_2S_2**	**Lhcb4.1-C_2S_2**
Magnification	22,500	22,500
Voltage(kV)	300	300
Electron exposure	60	60
Defocus range (µm)	-1 to -2	-1 to -2
Pixel size (Å)	1.06	1.06
Symmetry imposed	*C*2	*C*2
Initial particle images (no.)	1,722,401	872,009
Final particle images (no.)	128,938	68,270
Map resolution (Å)	3	3.1
FSC threshold	0.143	0.143
Map resolution range (Å)	2.8-7.4	2.8-7.1
**Model refinement**		
Initial model used (PDB code)	7OUI	7OUI
Model resolution (Å)	3.3	3.6
FSC threshold	0.5	0.5
**Model composition**		
Non-hydrogen atoms	71,222	70,362
Protein residues	6,886	6,872
Ligands	338	322
Map sharpening B factor ($Å^2$)	-68	-70.3
**B factors ($Å^2$)**		
Protein	54.19	68.74
Ligands	51.33	63.19
**R.m.s. deviations**		
Bond lengths (Å)	0.004	0.007
Bond angles (°)	1.068	1.296
**Validation**		
Molprobity score	1.7	1.77
Clashscore	7.72	8.33
Poor rotamers	0	0
**Ramachandran plot**		
Favored (%)	95.94	95.43
Allowed (%)	3.99	4.46
Disallowed (%)	0.07	0.12

Statistics for cryo-EM data collections, processing and structure refinement.

1. Sardar, S. *et al.* Molecular mechanisms of light harvesting in the minor antenna CP29 in near-native membrane lipidic environment. *Journal of Chemical Physics* **156**, 205101 (2022).

2. Mastronarde, D. N. SerialEM: A program for automated tilt series acquisition on Tecnai microscopes using prediction of specimen position. in *Microscopy and Microanalysis* vol. 9 1182–1183 (2003).

3. Wu, C., Huang, X., Cheng, J., Zhu, D. & Zhang, X. High-quality, high-throughput cryo-electron microscopy data collection via beam tilt and astigmatism-free beam-image shift. *J Struct Biol* **208**, (2019).

4. Punjani, A., Rubinstein, J. L., Fleet, D. J. & Brubaker, M. A. CryoSPARC: Algorithms for rapid unsupervised cryo-EM structure determination. *Nat Methods* **14**, 290–296 (2017).

5. Rohou, A. & Grigorieff, N. CTFFIND4: Fast and accurate defocus estimation from electron micrographs. *J Struct Biol* **192**, 216–221 (2015).

6. Pettersen, E. F. *et al.* UCSF Chimera - A visualization system for exploratory research and analysis. *J Comput Chem* **25**, 1605–1612 (2004).

7. Liebschner, D. *et al.* Macromolecular structure determination using X-rays, neutrons and electrons: Recent developments in Phenix. *Acta Crystallogr D Struct Biol* **75**, 861–877 (2019).

8. Emsley, P., Lohkamp, B., Scott, W. G. & Cowtan, K. Features and development of Coot. *Acta Crystallogr D Biol Crystallogr* **66**, 486–501 (2010).

9. Williams, C. J. *et al.* MolProbity: More and better reference data for improved all-atom structure validation. *Protein Science* **27**, 293–315 (2018).

10. Caffarri, S., Kouřil, R., Kereïche, S., Boekema, E. J. & Croce, R. Functional architecture of higher plant photosystem II supercomplexes. *EMBO Journal* **28**, 3052–3063 (2009).
