# Supplementary figures and images for: A stress-induced paralog of Lhcb4 controls the photosystem II functional architecture in Arabidopsis thaliana
Source: Nat Commun. 2025 Jul 26;16:6910. doi: 10.1038/s41467-025-62085-2 (PMC12297487; doi:10.1038/s41467-025-62085-2)

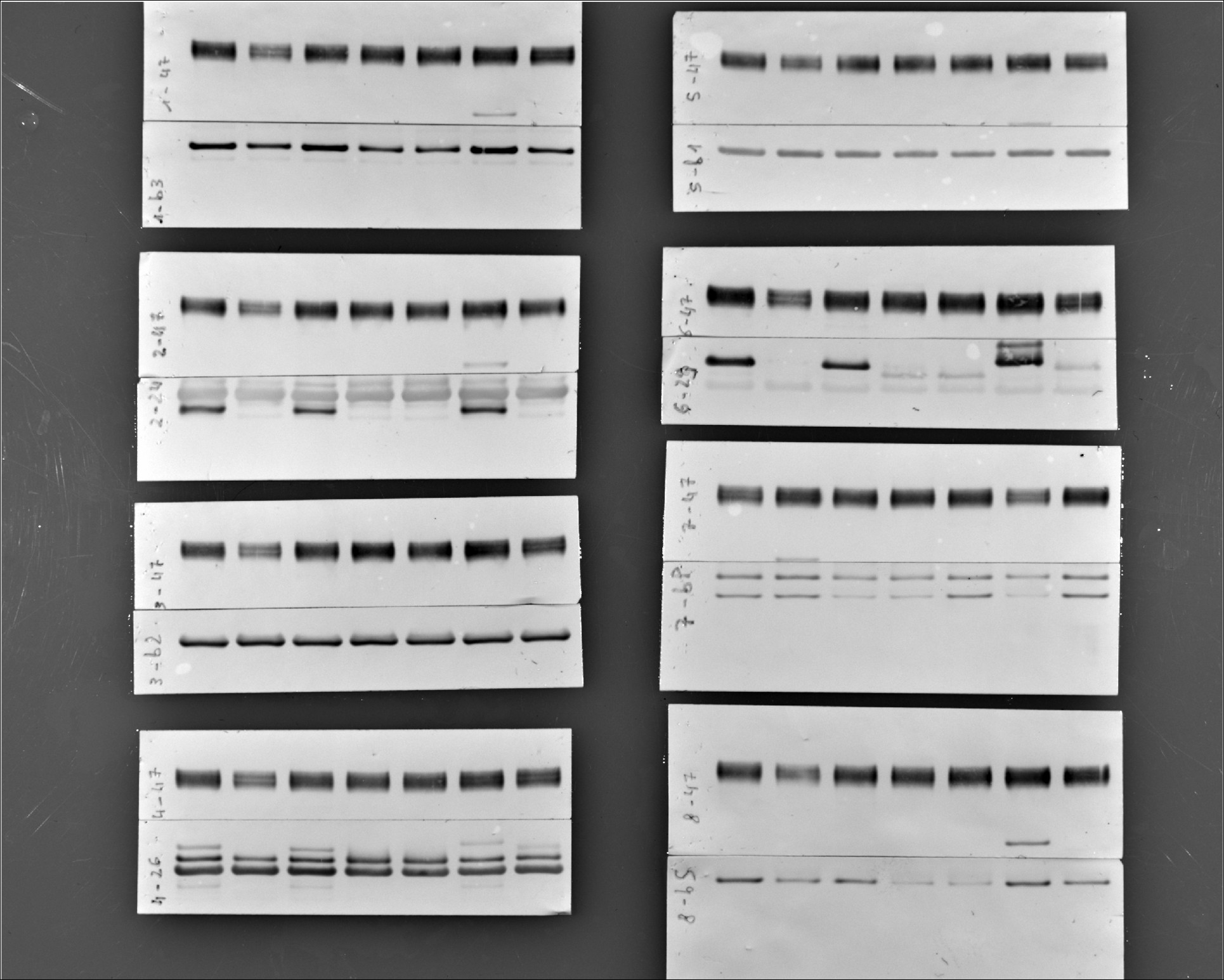

Supplement: Supplementary file 3 — Source Data file [file 41467_2025_62085_MOESM3_ESM.zip › Raw blot Fig. 1.jpg]

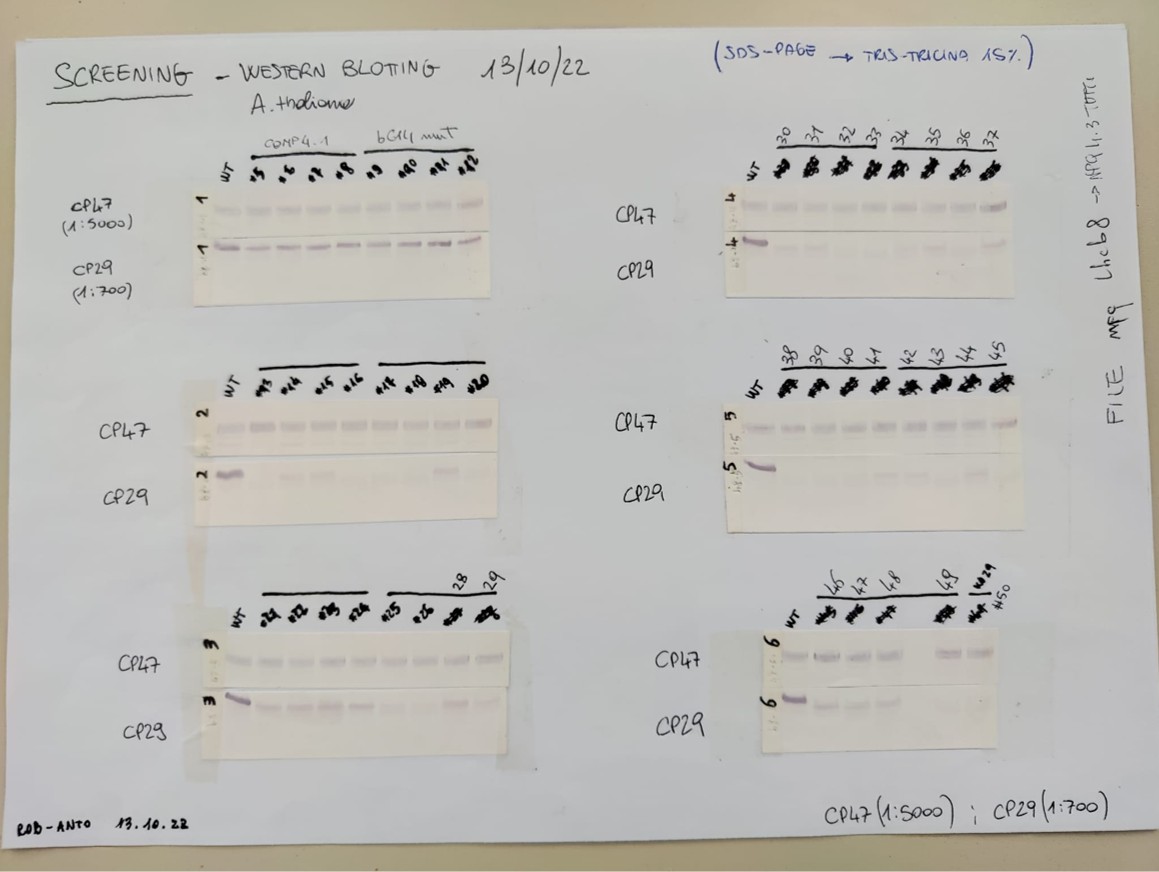

Supplement: Supplementary file 3 — Source Data file [file 41467_2025_62085_MOESM3_ESM.zip › Raw blot Fig. 4 and 6 (1).jpg]

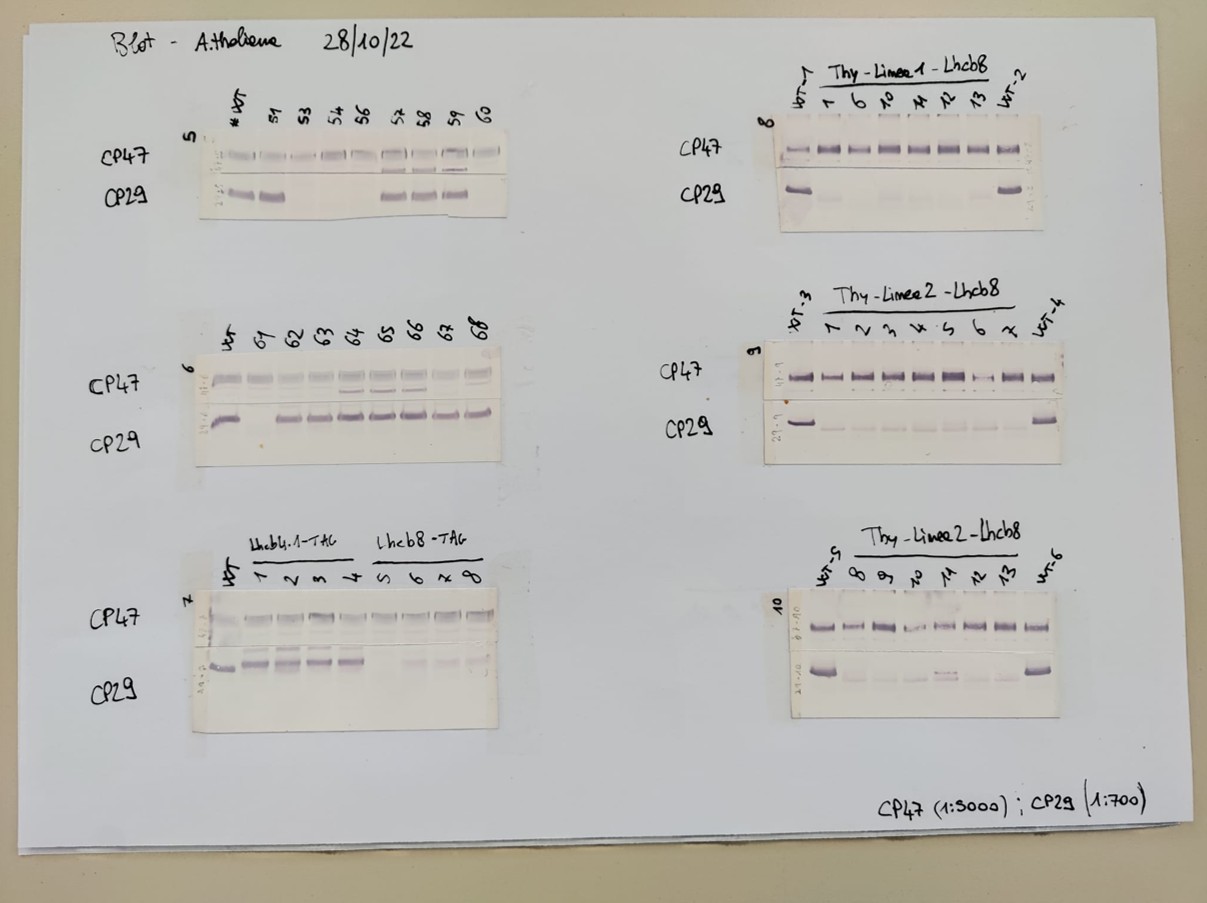

Supplement: Supplementary file 3 — Source Data file [file 41467_2025_62085_MOESM3_ESM.zip › Raw blot Fig. 4 and 6 (2).jpg]

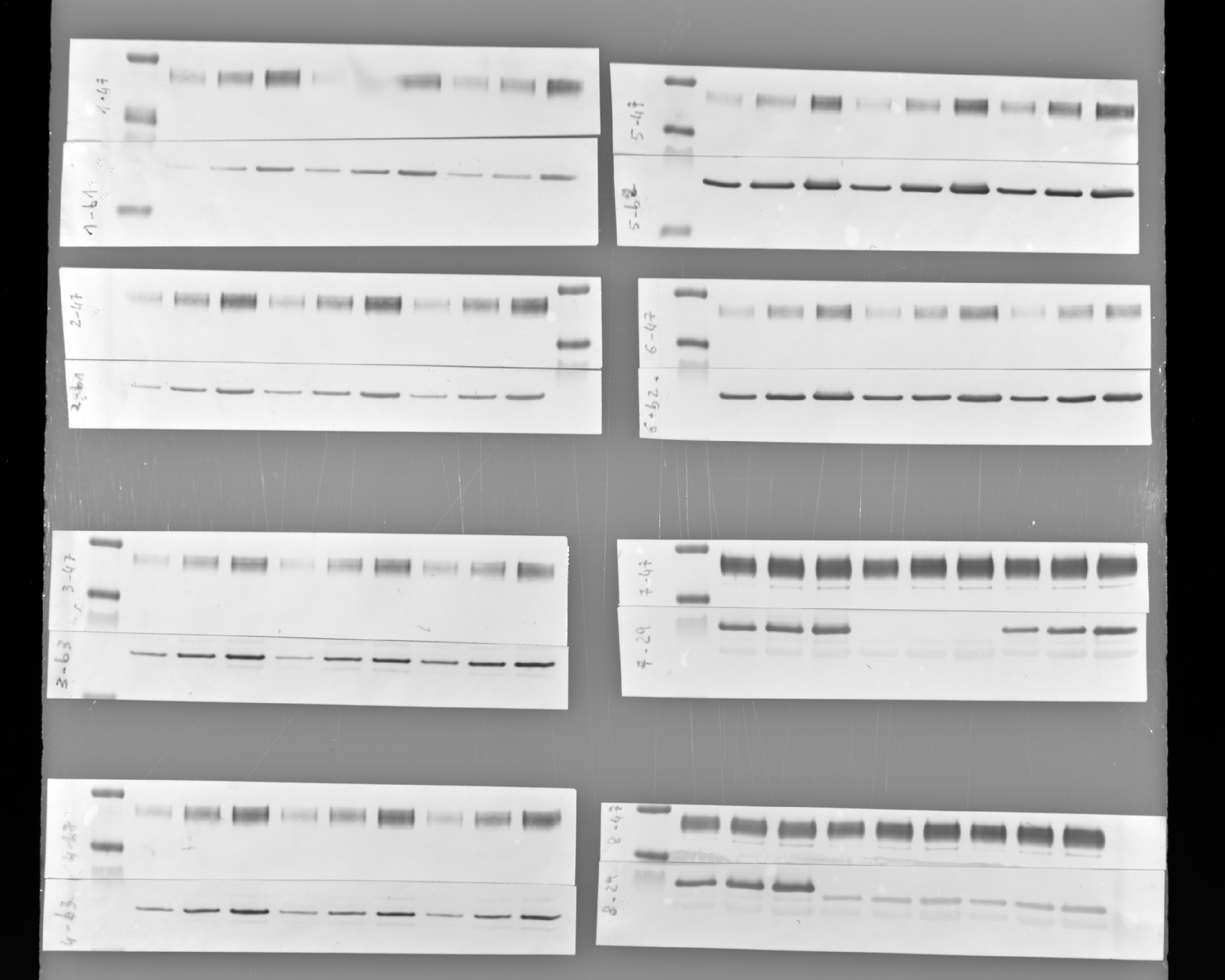

Supplement: Supplementary file 3 — Source Data file [file 41467_2025_62085_MOESM3_ESM.zip › Raw blot Suppl Fig. 3 (1).jpg]

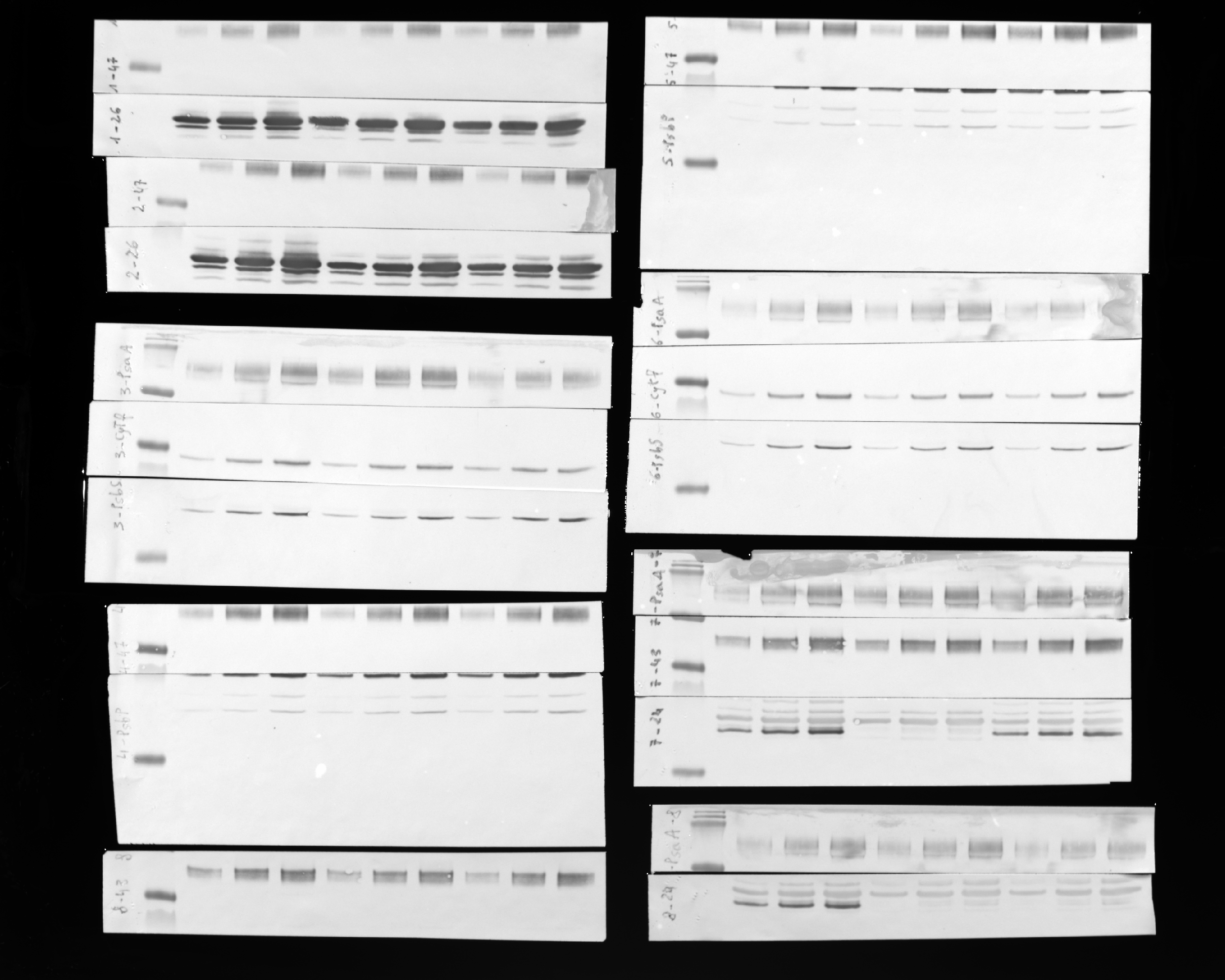

Supplement: Supplementary file 3 — Source Data file [file 41467_2025_62085_MOESM3_ESM.zip › Raw blot Suppl Fig. 3 (2).jpg]

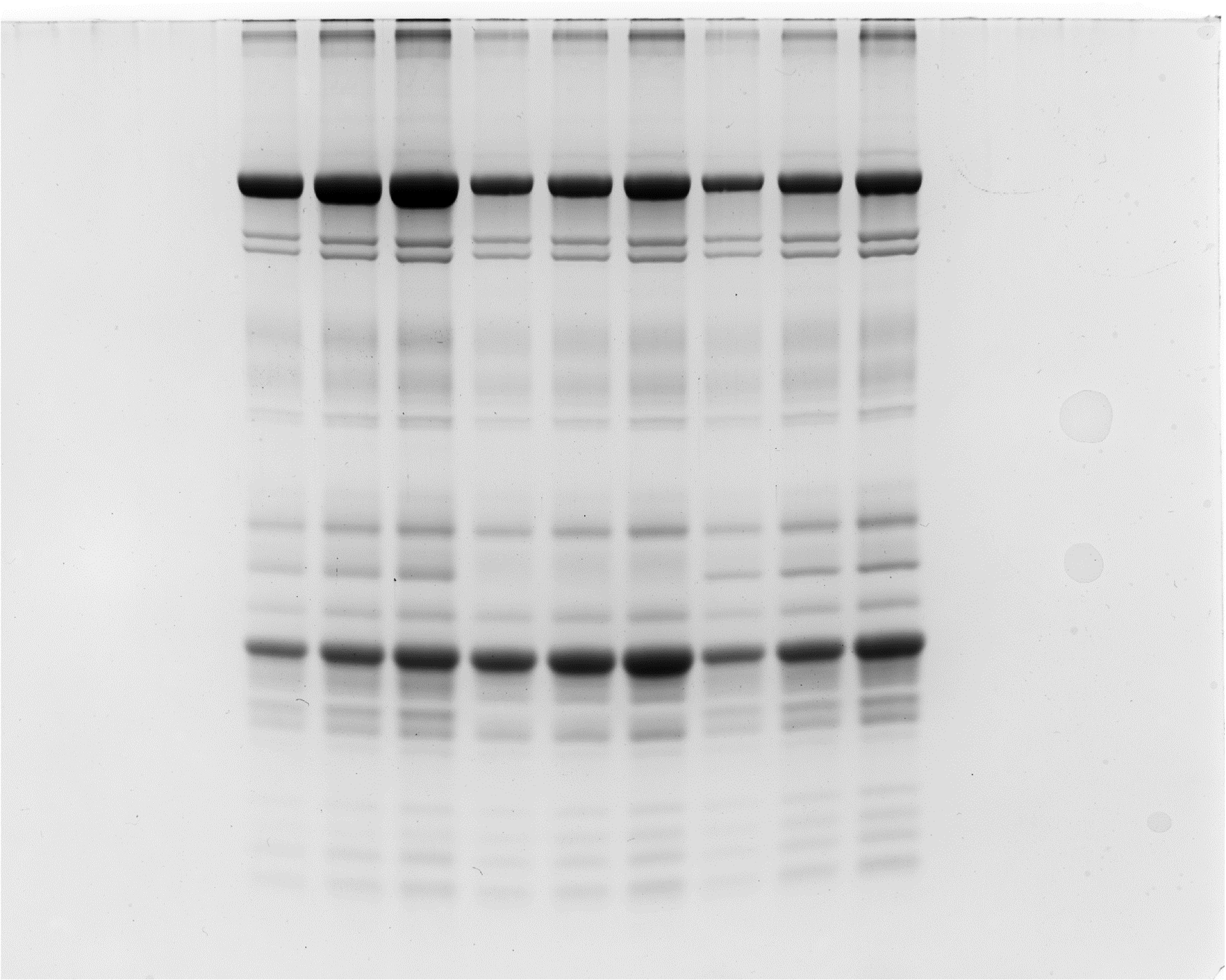

Supplement: Supplementary file 3 — Source Data file [file 41467_2025_62085_MOESM3_ESM.zip › Raw gel Suppl Fig. 4 (1).jpg]

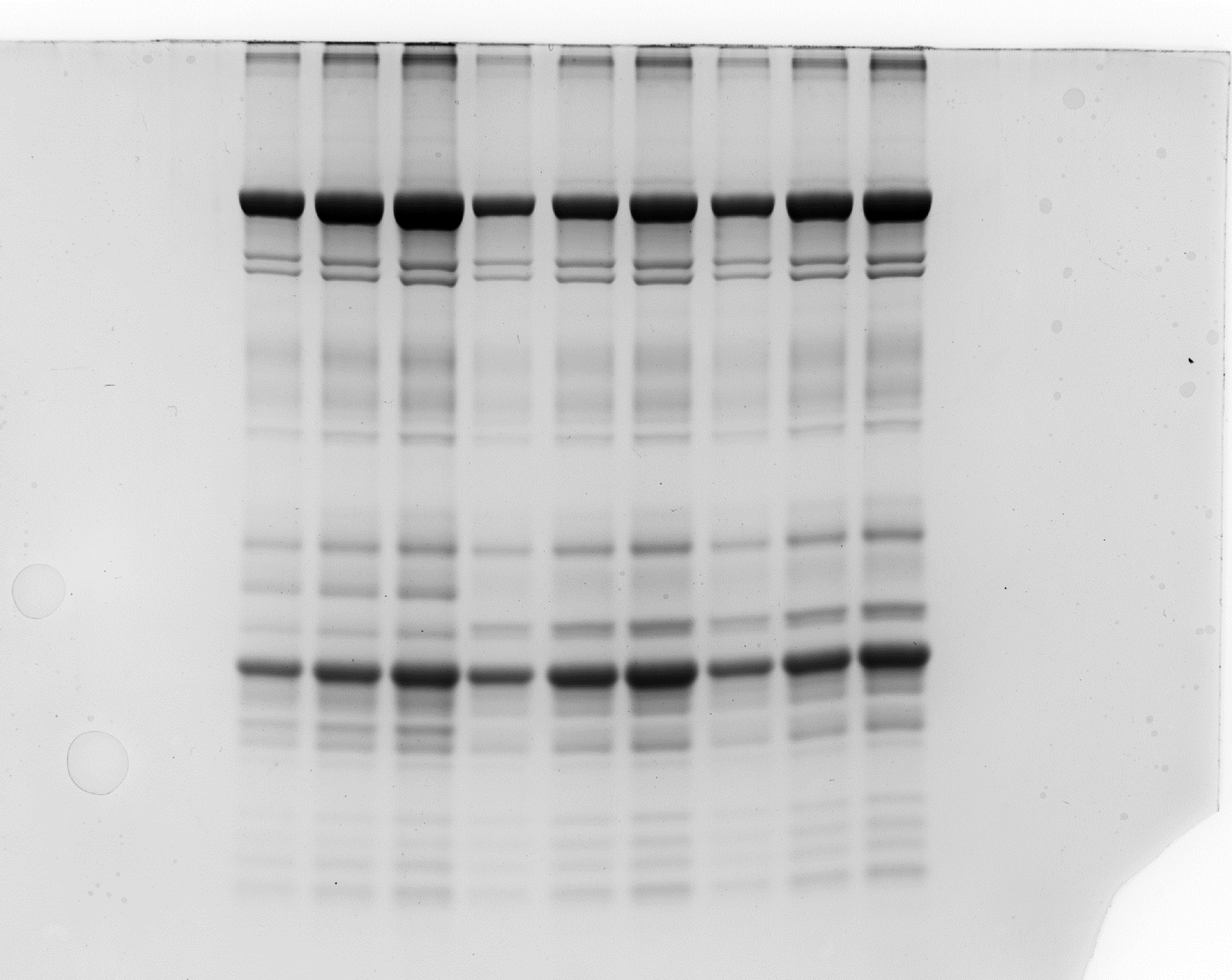

Supplement: Supplementary file 3 — Source Data file [file 41467_2025_62085_MOESM3_ESM.zip › Raw gel Suppl Fig. 4 (2).jpg]
